# *Dichodon parvipetalum*, a new combination for the Chinese Caryophyllaceae

**DOI:** 10.3897/phytokeys.69.8494

**Published:** 2016-09-09

**Authors:** Gang Yao

**Affiliations:** 1 Key Laboratory for Plant Diversity and Biogeography of East Asia, Kunming Institute of Botany, Chinese Academy of Sciences, Kunming 650210, China; 2 Plant Germplasm and Genomics Center, Germplasm Bank of Wild Species, Kunming Institute of Botany, Chinese Academy of Sciences, Kunming, 650201, China

**Keywords:** Caryophyllaceae, Cerastium, Dichodon, Dichodon parvipetalum, China

## Abstract

The generic name *Dichodon* (Bartl. ex Rchb.) Rchb. was previously reinstated based on results from recent molecular phylogenetic studies. Accordingly, *Dichodon parvipetalum* (Hosok.) G. Yao, a new combination for the species *Cerastium parvipetalum* Hosok. is proposed.

## Introduction

The genus *Cerastium* L. in the broad sense consists of nearly 100 species mainly distributed in temperate and cold regions, with 23 species occurring in China (Lu and Morton 2001). Traditionally, the genus was subdivided into two subgenera: *Cerastium* (characterized by five carpels, five styles and ten capsule teeth) and *Dichodon* (Bartl. ex Rchb.) Boiss. (characterized by three carpels, three styles and six capsule teeth) (Schischkin 1970). In a recent molecular phylogenetic study (Greenberg and Donoghue 2011), the subgenus Cerastium was supported as the sister-group of *Moenchia* Ehrhart., a clade with four styles and eight capsule teeth, while the other subgenus Dichodon was sister to *Holosteum* L., which has three styles and six capsule teeth. Based on these results, Hernández-Ledesma et al. (2015) accepted the subgenus Dichodon at the generic level and reinstated it as *Dichodon* (Bartl. ex Rchb.) Rchb. in their comprehensive taxonomic study of Caryophyllales. In the *Flora of China*, two species (viz. *Cerastium cerastoides* (L.) Britton and *Cerastium parvipetalum* Hosok.) belonging to the subgenus Dichodon were accepted (Lu and Morton 2001). The former species has already been transferred to *Dichodon* (*Dichodon cerastoides* (L.) Rchb.) by Reichenbach (1841), while the other species, endemic in Taiwan, has not been formally transferred to *Dichodon*. Accordingly, the new combination *Dichodon parvipetalum* is proposed below.

## New combination

### Dichodon parvipetalum

Taxon classificationPlantaeCaryophyllalesCaryophyllaceae

(Hosok.) G. Yao comb. nov.

urn:lsid:ipni.org:names:60473146-2

#### Basionym.

*Cerastium parvipetalum* Hosok., Trans. Nat. Hist. Soc. Taiwan 22: 227. 1932. TYPE: CHINA. Taiwan, Kaohsiung, 3 Jan. 1931, *T. Hosokawa 93* (Holotype: TAI!, no.116065)

## Supplementary Material

XML Treatment for Dichodon parvipetalum
